# Annotations

**Published:** 1902-01-11

**Authors:** 


### Annotations.

### The King and the Consumptives.

While expressing our admiration of the generosity of Sir Ernest Cassel in presenting to the King the munificent sum of £200,000 to be devoted to some philanthropic purpose, we may properly add that the country is additionally indebted to him for the manner in which the gift has been made in that it has caused us to receive yet another proof of His Majesty's practical sympathy with suffering and want. Nothing could be more appropriate to present day needs than the King's action in devoting this large sum of money to the erection of a sanatorium for tuberculosis, and nothing we think could more definitely show how thoroughly he has grasped the difficulties and uncertainties which surround the whole problem of the control of tuberculosis than the way in which, rather than throw the spending of this princely gift into the hands of established authorities, he has asked the help of all medical men of whatever nationality, and has offered such money prizes for essays and plans as are sure to prove attractive to the most competent. From the wording of the announcement it appears that of the 100 beds which are to be provided under the scheme, 88 are to be assigned "to the more necessitous classes, whilst 12 will be reserved for the well-to-do." We like that phrase, "the more necessitous classes," which we presume to mean "more necessitous" than "the well-to-do," and in it we see another proof of wisdom, for it would be disastrous were a great scheme like this to be diverted into a mere plan for the saving of the rates. Nothing would be further from our wish than to do anything to debar paupers from any advantages which any mode of treatment may offer. But the indigent population have their own proper guardians who are bound to supply them with what is medically necessary, and the consumptive paupers' best chance of obtaining the benefits of sanatorium treatment lies in such treatment becoming recognised among other classes as medically necessary. It is the great lower middle-classes that most require help in this matter. When once the sanatorium treatment of consumption takes such root among them as to be regarded as essential, the guardians will have to provide it for those under their care just as they have to provide food, shelter, and other necessaries.

### Universal Military Service.

The literary topic of the week has no doubt been the publication of "The Islanders" by Rudyard Kipling. This has appealed to many and disgusted some—with all of which we have nothing to do. But the teaching of the poem and the logical outcome of the spirit breathed therein, namely, universal military service, is a matter of much interest from medical and sanitary points of view. To make a very perfect soldier it is probable that continuous service in the ranks for a considerable time is necessary; but, in view of our large foreign possessions, which we could hardly hope to garrison with compulsory service troops, it seems not unlikely that if it should ever be decided to enforce universal service the plan adopted would be based on our militia system, and we cannot but see what enormous hygienic benefits such a plan might be made to confer upon the working classes of this country. Every year the people are drifting more and more towards the towns. Not only are the towns absorbing the manhood of the nation, but more especially are they absorbing the young, and it becomes a very serious problem how to maintain and develop the stamina of the race under such conditions. The deteriorating influences of town life may at least be partially neutralised by country holidays. It is not entirely pleasure seeking that drives so many thousands to country and seaside every summer, but rather the satisfaction of an instinct, and one cannot but see that to be "up with the militia" for a time every year would give to a large and a growing proportion of the young men in this country exactly the sort of outdoor holiday which is wanted to counteract in some degree the bad effects of their crowded life in towns. No one who has watched the improvement which takes place during the six weeks of training in the health and good looks of those regiments of militia which are largely recruited from mining and manufacturing populations, can feel any doubt as to the great advantages of that training from a hygienic point of view.

### "Bad Arms."

It is hardly to be doubted that vaccination is being seriously discredited by the prevalence of "bad arms." Not only does the treatment of these unfortunate results become a considerable item in the daily work of many practitioners, but the more or less futile attempts to soothe the ruffled feelings of his disgusted patients becomes in some cases a very serious tax upon the doctor's time as well as, perhaps, upon his inventive faculties. It is one of the penalties we pay for having popularised medicine. Patients nowadays think they know all about germs and antiseptics, and at least they know sufficient to ask some very awkward questions. When a man who has his living to earn finds himself, as a result of re-vaccination, not only rendered very uncomfortable but to a large extent incapacitated—with his arm in a sling, swollen, inflamed and useless—he is apt to say at once, and with no small emphasis, that the fault must lie between the doctor and the lymph; and as we none of us like to take blame upon ourselves while we can hardly dispute the logic of his position we are afraid that it too often happens that the onus is thrown upon the vaccine. This may be right or it may be wrong, but anyway the effect is the same, namely, to discredit vaccination. What we now speak of is not merely an affair between doctor and patient. In every place where men and women congregate we hear the same thing discussed. "Bad arms" and how to avoid them are universal topics of conversation, and it is hardly to be wondered at that certain ingenuous people who go in for short views of things avoid them by the simple plan of avoiding vaccination altogether. There is no doubt that the publicity given to the possession of a "bad arm" by sling, by tapes, and by general grumbling, throws great difficulty in the way of controlling the spread of small-pox, and that it would be worth much to be able to do secondary vaccination with something like assured confidence that one would not cripple one's patient.

